# Resveratrol Mitigates Inflammation by Modulating Tumor Necrosis Factor-Alpha Receptors (TNFRs) in a 2,4,6-Trinitrobenzene Sulfonic Acid (TNBS)-Induced Rat Model of Colitis

**DOI:** 10.3390/ijms26125779

**Published:** 2025-06-16

**Authors:** Médea Veszelka, József Hegyközi, Nikoletta Almási, Szilvia Török, Bence Pál Barta, Izabella Nagy, Denise Börzsei, Nikolett Bódi, Mária Bagyánszki, Renáta Szabó, Csaba Varga

**Affiliations:** 1 Department of Physiology, Anatomy, and Neuroscience, Faculty of Science and Informatics, University of Szeged, 6726 Szeged, Hungary; almasi@expbio.bio.u-szeged.hu (N.A.); tszilvia@bio.u-szeged.hu (S.T.); barta.bence@bio.u-szeged.hu (B.P.B.); nagiza@expbio.bio.u-szeged.hu (I.N.); denise@expbio.bio.u-szeged.hu (D.B.); bodi.nikolett@bio.u-szeged.hu (N.B.); bmarcsi@bio.u-szeged.hu (M.B.); szrenata@bio.u-szeged.hu (R.S.); vacs@bio.u-szeged.hu (C.V.); 2 HR-Pharma Ltd., 6726 Szeged, Hungary; hegykozi.jozsef@expbio.bio.u-szeged.hu; 3 Department of Microbiology, Faculty of Science and Informatics, University of Szeged, 6726 Szeged, Hungary

**Keywords:** resveratrol, TNBS-induced colitis, oxidative stress, colon inflammation, pro-inflammatory cytokine, TNF-alpha, TNFR-α receptor, IBD

## Abstract

Several substances with antioxidant and anti-inflammatory properties are currently being investigated as potential adjunctive or standalone treatments for inflammatory bowel disease (IBD). One such substance is resveratrol (RES), also known as 3,5,4′-trihydroxy-trans-stilbene, a natural dietary polyphenol with diverse health-promoting effects. In this study, male Wistar–Hannover rats received oral RES supplementation at doses of 5, 10, or 20 mg/kg/day for 28 days. On day 25 colitis was induced using intracolonic administration of 2,4,6-trinitrobenzene sulphonic acid (TNBS). Based on histological and planimetric analysis, the 10 mg/kg dose significantly reduced colonic ulceration and pro-inflammatory cytokine tumor necrosis factor-alpha (TNF-α) expression compared to the TNBS group. Immunohistochemistry also revealed that RES at this dose attenuated the intensity of TNF-α receptors, namely TNFR1 and TNFR2. Furthermore, the concentration of lipocalin-2 (Lcn-2) was significantly elevated in TNBS-induced colitis. In conclusion, our findings suggest that RES may exert its protective effects partly through the modulation of TNF receptor signaling in TNBS-induced colitis.

## 1. Introduction

Inflammatory bowel disease (IBD) is a global disease of the 21st century that consists of two major forms ulcerative colitis (UC) and Crohn’s disease (CD) [1]. The increasing incidence of IBD, affecting over 2 million individuals in Europe, is attributed to genetic susceptibility, impaired immune responses, and environmental risk factors, such as smoking, poor diet, and the consumption of certain medicines [2,3,4]. The main differences between UC and CD are the localization of the disease and the severity of inflammation. UC is characterized by mucosal inflammation initiating in the colon and rectum lining, causing ulcers to be limited to the mucosa [5,6]. However, CD usually appears in patches through the entire gastrointestinal tract, and inflammation extends to all layers of the intestinal wall [6,7].

Tumor necrosis factor-alpha (TNF-α) is a key mediator of several inflammatory pathways, such as the inducible nitric oxide synthase pathway. TNF-α binds two cell surface receptors, TNFR1 and TNFR2, both of which are implicated in the pathogenesis of IBD, and their expression is well correlated with disease activity. TNFR1 is ubiquitously expressed across almost all cell types, whereas TNFR2 expression is largely restricted to specific subpopulations of T lymphocytes, endothelial cells, and certain neuronal and glial cells [8]. TNFR1 contains an intracellular death domain that triggers apoptotic and degenerative pathways [9,10], while TNFR2 is associated with cell survival and tissue regeneration, mainly via activation of the nuclear factor-kappa B (NF-κB) and the phosphoinositide 3-kinase/protein kinase B signaling pathways [11,12]. Increasing evidence suggests that selective activation or modulation of TNFR2 could offer therapeutic benefits in inflammatory diseases, including IBD, by promoting mucosal healing while minimizing systemic immunosuppression [13].

Furthermore, lipocalin-2 (Lcn-2), also known as neutrophil gelatinase-associated lipocalin (NGAL), is a biomarker shown to be expressed in normal tissues and released at sites of inflammation [14,15]. Several studies have shown that the expression and secretion of Lcn-2 can be induced by TNF-α [16,17]. Furthermore, the inductive effect of TNF-α on Lcn-2 messenger RNA expression is dependent on NF-κB pathway, since small interfering RNA -mediated knockdown of the p65 subunit of NF-κB significantly attenuates Lcn-2 expression and secretion.

Various natural molecules are considered potential agents to treat inflammatory disorders [18]. Among them, resveratrol (RES) (3,5,4′-trihydroxy-trans-stilbene) is a naturally occurring polyphenol found in a large number of various plant species such as peanuts, grapes, and mulberries [19] and was identified as a component of red wine in 1992 [20]. RES has been reported to exhibit neuroprotective [21], antioxidant [22], anti-tumor, and anti-inflammatory properties [23]. Therefore, RES may be effective in the treatment of colitis due to its anti-inflammatory effects. One of the most commonly used animal models to study IBD is the 2,4,6-trinitrobenzene sulfonic acid (TNBS) model [24,25]. The protective effect of RES has been demonstrated in TNBS colitis [26], but the exact mechanism of the protection remains to be elucidated. Therefore, we aimed to study the effects of long-term RES supplementation in experimental IBD. To confirm the protective effects of RES in colonic inflammation, we performed histological and immunohistochemical analyses. Furthermore, through biochemical measurements, we evaluated the levels of Lcn-2 and TNF-α as disease activity markers. Our results reveal that the four weeks of RES treatment at a concentration of 10 mg/kg/day suppressed the expression of pro-inflammatory cytokine tumor necrosis factor-alpha (TNF-α) and modulated the expression of its receptors, TNFR1 and TNFR2, suggesting a multi-target anti-inflammatory mechanism. To the best of our knowledge, this is the first study to comprehensively assess the colonic expression profile of TNFRs in a TNBS-induced rat model of IBD subjected to RES treatment.

## 2. Results

### 2.1. Effects of RES Supplementation on the Severity of Inflammation in TNBS Colitis

**Figure 1:**
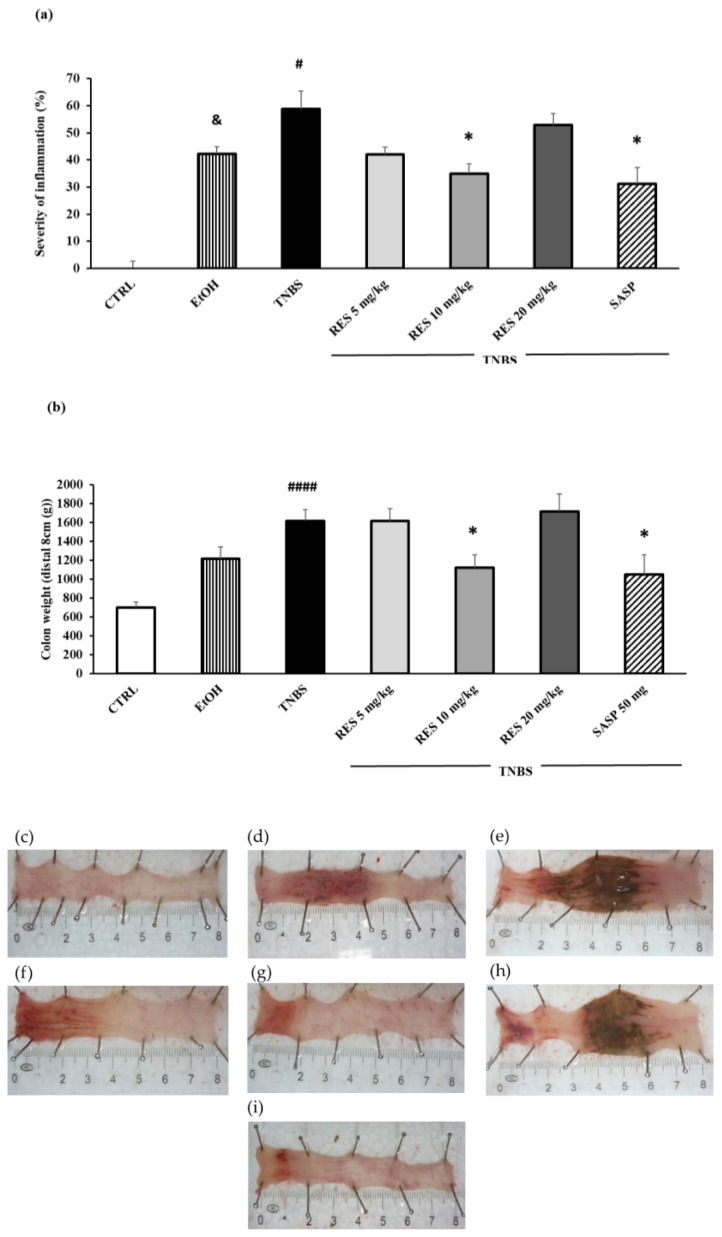
(**a**) Effect of resveratrol (RES) on the severity of inflammation and (**b**) colon weight in 2,4,6-trinitrobenzene sulfonic acid (TNBS)-induced colitis. Representative images of the colonic damage: (**c**) CTRL (no treatment); (**d**) 50% ethanol (EtOH enema); (**e**) TNBS enema; (**f**) TNBS + RES 5 mg/kg; (**g**) TNBS + RES 10 mg/kg; (**h**) TNBS + RES 20 mg/kg; (**i**) TNBS + sulfasalazine (SASP). Data represented by mean ± S.E.M.; n = 4–12/group; statistical significance: * *p* < 0.05 TNBS vs. TNBS + treatment; # *p* < 0.05 and #### *p* < 0.001 CTRL vs. TNBS; and & *p* < 0.05 CTRL vs. EtOH.

Colitis was induced in rats using TNBS. Macroscopic colonic injuries were analyzed 72 h later. The percentage of the macroscopically visible mucosal injury was expressed as the percentage of the total colonic segmented area (Figure 1a). Moreover, we analyzed the weight of the colon and found this was significantly increased in the TNBS-treated rats compared to the CTRL group (Figure 1b). The dissolvent of TNBS, EtOH, caused marked inflammation and ulceration in the colon compared to the healthy control (Figure 1d). However, the administration of TNBS caused a much more pronounced ulceration of the colon. The 5 (Figure 1f) and 20 mg/kg (Figure 1h) doses of RES did not affect significantly the colonic inflammation compared to TNBS. As was observed pre-treatment with 10 mg/kg, RES significantly diminished inflammation compared to TNBS group (Figure 1g) (34.92 ± 3.64 vs. 58.72 ± 6.64%), an effect which was similar to our positive control. As a positive control, SASP (50 mg/kg/day orally) significantly attenuated the severity of the colonic inflammation (Figure 1i) compared to the TNBS group (Figure 1e) (31.15 ± 6.07 vs. 58.72 ± 6.64%). Representative images of the colons are shown in Figure 1c–i.

### 2.2. Histological Results of Haematoxylin and Eosin Staining

**Figure 2:**
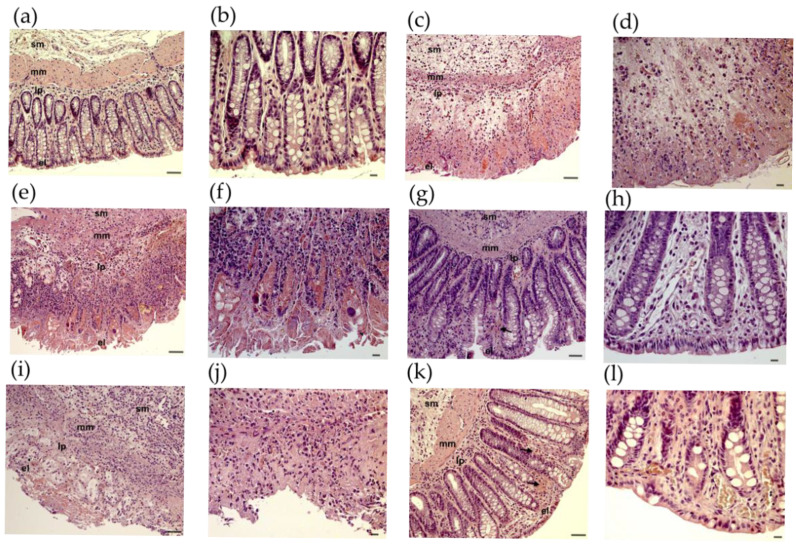
Histopathological changes in rat colon in 2,4,6-trinitrobenzene sulfonic acid (TNBS) model of colitis. Representative images of rat colon after haematoxylin and eosin staining: (**a**,**b**) CTRL (no treatment); (**c**,**d**) TNBS enema; (**e**,**f**) TNBS enema + resveratrol (RES) 5 mg/kg; (**g**,**h**) TNBS + RES 10 mg/kg; (**i**,**j**) TNBS + RES 20 mg/kg; (**k**,**l**) TNBS + sulfasalazine. Arrow: hemorrhage sm: submucosa, mm: muscularis mucosae, lp: lamina propria, el: epithelial layer. Scales: 50 μm (**a**,**c**,**e**,**g**,**i**,**k**), 20 μm (**b**,**d**,**f**,**h**,**j**,**l**).

On examination of the colon from CTRL rats, the histological features were typical of a normal structure. There were no signs of inflammation or hemorrhages (Figure 2a,b). However, in the TNBS-treated rats, severe histological changes were observed (Figure 2c,d), such as distorted crypts, hemorrhages, and infiltrated white blood cells. In these sections, the inflammation was extended through both the mucosa and submucosa.

At a concentration of 5 mg/kg, RES treatment had no significant alleviating effect on TNBS-induced inflammatory changes (Figure 2e,f). After administration of RES in the 10 mg/kg dose, there was a considerable attenuation of morphological signs of cell damage (Figure 2g,h) and a mild to moderate grade of inflammation characterized by hemorrhages. RES at 20 mg/kg did not appear to be more effective (Figure 2i,j).

Although the application of SASP had a significant beneficial effect, it could not fully prevent the harmful effects of TNBS, as colonic hyperemia was still observed (Figure 2k,l).

The magnifications for pathological images are 200× (Figure 2a,c,e,g,i,k) and 400× (Figure 2b,d,f,h,j,l).

### 2.3. The Effects of RES Treatment on the Colonic TNF-α Concentration

**Figure 3:**
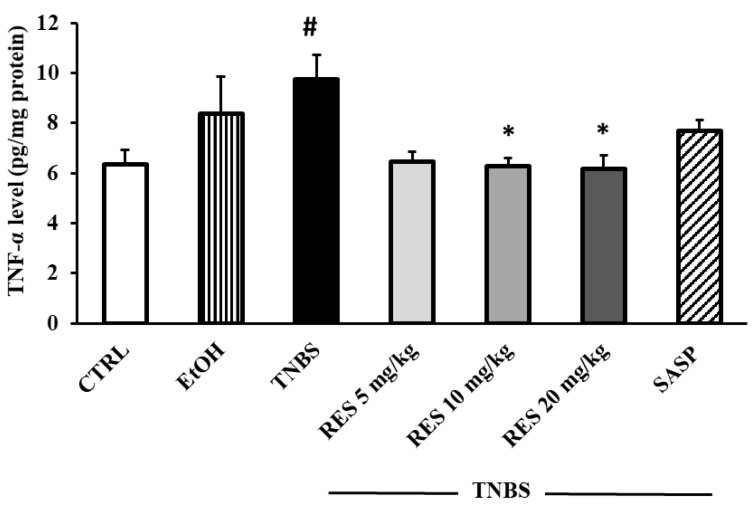
The effects of resveratrol (RES) supplementation on colonic tumor necrosis factor-alpha concentration (TNF-α, expressed as pg/mg protein) in 2,4,6-trinitrobenzene sulfonic acid (TNBS)-induced rat colitis. CTRL (no treatment); EtOH (50% ethanol, the solvent of TNBS); TNBS enema; TNBS + RES 5 mg/kg; TNBS + RES 10 mg/kg; TNBS + RES 20 mg/kg; TNBS + sulfasalazine (SASP). Results are presented as mean ± S.E.M.; n = 4-6/group; statistical significance: * *p* < 0.05 TNBS vs. TNBS + treatment; # *p* < 0.05 CTRL vs. TNBS.

To investigate the effects of RES on inflammatory status, we determined the colonic TNF-α concentration. TNF-α levels were significantly higher in the TNBS group than in the CTRL group (6.36 ± 0.55 vs. 9.75 ± 0.95 pg/mg protein). 10 mg/kg and 20 mg/kg RES pre-treatment caused a significant reduction in TNF-α concentrations compared to the TNBS groups (6.26 ± 0.34 and 6.17 ± 0.53 vs. 9.75 ± 0.95 pg/mg protein). In the 5 mg/kg RES-treated group we also found a marked decrease in TNF-α level (Figure 3).

### 2.4. TNF-α, TNFR1, and TNFR2 Fluorescent Immunohistochemistry

**Figure 4:**
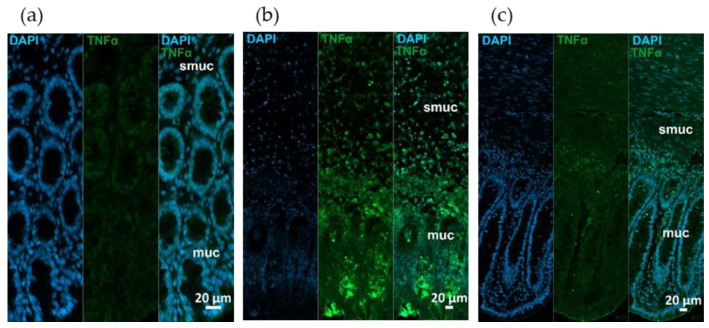
Representative fluorescent micrographs of paraffin sections from (**a**) control, (**b**) 2,4,6-trinitrobenzene sulfonic acid (TNBS)-treated, and (**c**) TNBS + resveratrol (RES) 10 mg/kg rats showing different intestinal layers after TNF-α immunohistochemistry. Nuclei were counterstained with DAPI (blue). muc—mucosa, smuc—submucosa, scale bars: 50 µm.

Fluorescent immunohistochemistry revealed TNF-α and TNFRs immunoreactivity in different intestinal layers on paraffin sections. Strong TNF-α immunoreactivity was observed in rats with TNBS-induced colitis, while it was moderated in 10 mg/kg dose of RES pre-treated group relative to very low TNF-α intensity in control samples (Figure 4).

**Figure 5:**
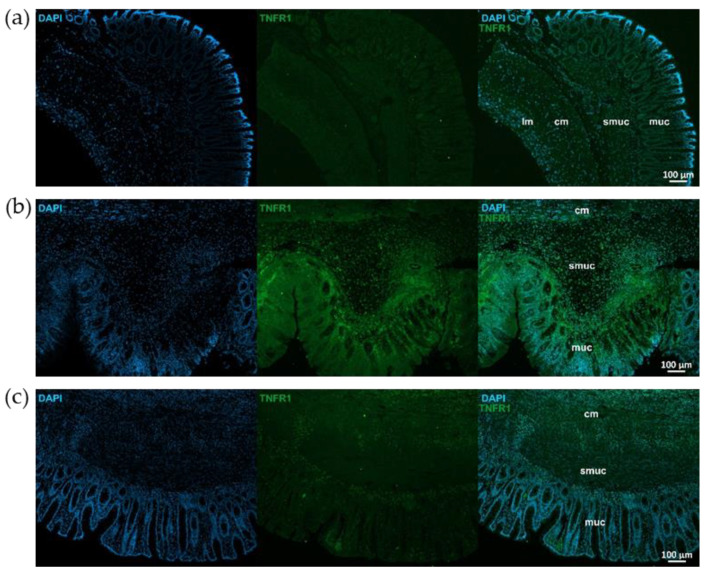
Representative fluorescent micrographs of paraffin sections from control (**a**), 2,4,6-trinitrobenzene sulfonic acid (TNBS)-treated (**b**), and TNBS + resveratrol (RES) 10 mg/kg (**c**) rats showing different intestinal layers after TNFR1 immunohistochemistry. Nuclei were counterstained with DAPI (blue). muc—mucosa, smuc—submucosa, cm—circular smooth muscle, lm—longitudinal smooth muscle, scale bars: 100 µm.

**Figure 6:**
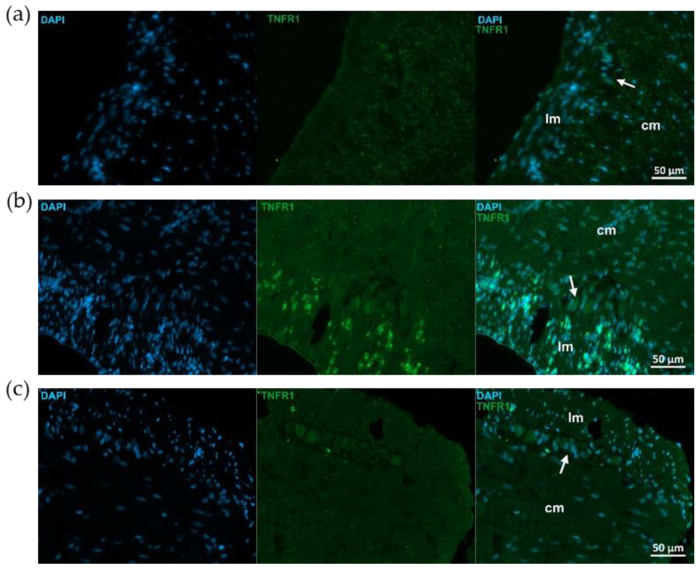
Representative fluorescent micrographs of paraffin sections from control (**a**), 2,4,6-trinitrobenzene sulfonic acid (TNBS)-treated (**b**), and TNBS + resveratrol (RES) 10 mg/kg (**c**) rats showing different intestinal layers after TNFR1 immunohistochemistry. Nuclei were counterstained with DAPI (blue). cm—circular smooth muscle, lm—longitudinal smooth muscle, arrows—myenteric ganglia, scale bars: 50 µm.

Similar immunostaining was described after TNFR1 immunohistochemistry, showing intensified TNFR1 labeling in the mucosa and submucosa of TNBS group, attenuated intensity in 10 mg/kg dose of RES group, and weak TNFR1 immunoreactivity in control rats (Figure 5). However, the pattern of TNFR1 immunostaining was not the same in all gut layers. In the longitudinal smooth muscle of TNBS-treated rats, enhanced immunoreactivity was visible compared to the control or 10 mg/kg dose of RES groups, while it was not observed in the circular muscle layer (Figure 6).

**Figure 7:**
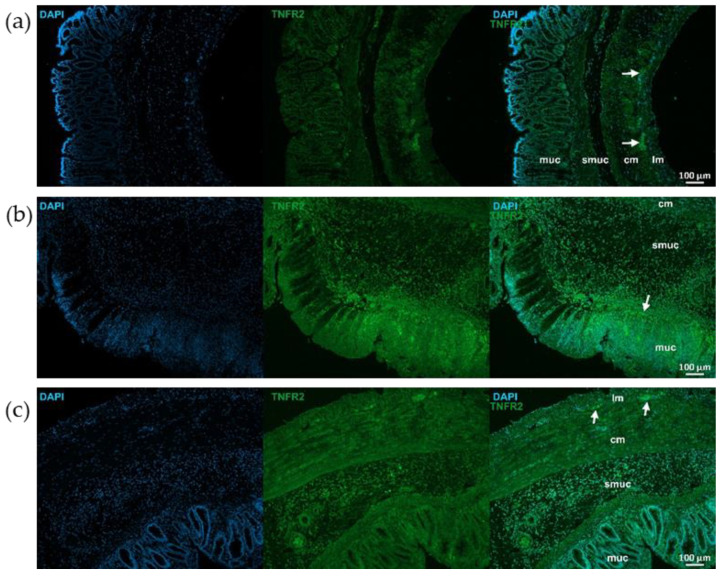
Representative fluorescent micrographs of paraffin sections from control (**a**), 2,4,6-trinitrobenzene sulfonic acid (TNBS)-treated (**b**), and TNBS + resveratrol (RES) 10 mg/kg (**c**) rats showing different intestinal layers after TNFR2 immunohistochemistry. Nuclei were counterstained with DAPI (blue). muc—mucosa, smuc—submucosa, cm—circular smooth muscle, lm—longitudinal smooth muscle, arrows—myenteric ganglia, scale bars: 100 µm.

**Figure 8:**
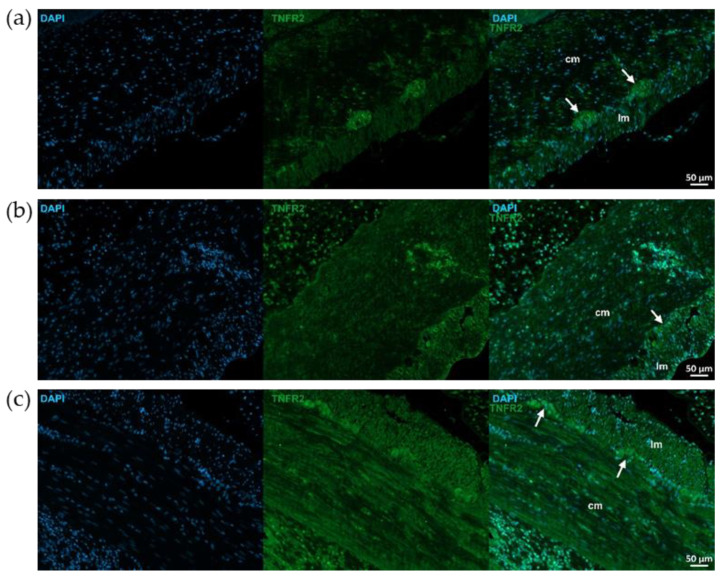
Representative fluorescent micrographs of paraffin sections from control (**a**), 2,4,6-trinitrobenzene sulfonic acid (TNBS)-treated (**b**), and TNBS + resveratrol (RES) 10 mg/kg (**c**) rats showing different intestinal layers after TNFR2 immunohistochemistry. Nuclei were counterstained with DAPI (blue). cm—circular smooth muscle, lm—longitudinal smooth muscle, arrows—myenteric ganglia, scale bars: 50 µm.

TNFR2 immunoreactivity was stronger than TNFR1 in control animals, particularly in myenteric ganglia (Figure 7 and Figure 8). TNFR2 labeling intensity was more powerful in the mucosa, submucosa and also muscular layers of the TNBS group and moderated in the RES group (Figure 7 and Figure 8).

### 2.5. Measurement of Colonic Lipocalin-2 Concentration

**Figure 9:**
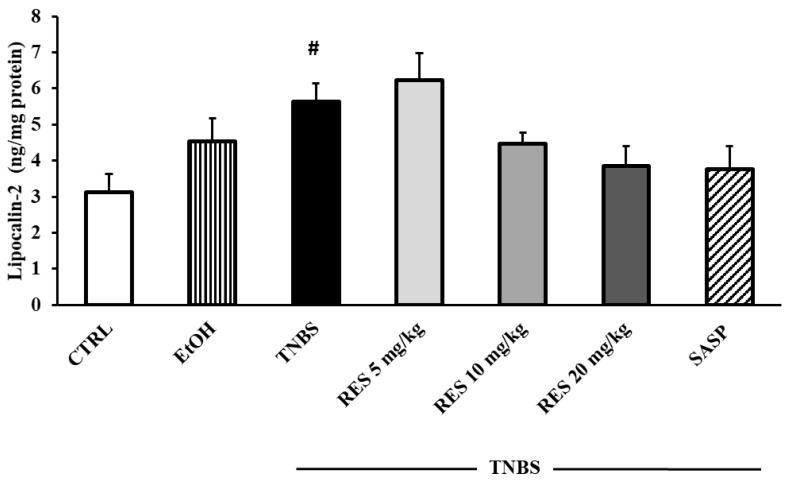
The effects of resveratrol (RES) on colonic lipocalin-2 concentration (Lcn-2, expressed as ng/mg protein) in 2,4,6-trinitrobenzene sulfonic acid (TNBS)-induced rat colitis. CTRL (no treatment); EtOH (50% ethanol, the solvent of TNBS); TNBS enema; TNBS + RES 5 mg/kg; TNBS + RES 10 mg/kg; TNBS + RES 20 mg/kg; TNBS + sulfasalazine (SASP). Results are presented as mean ± S.E.M.; n = 4–8/group; statistical significance: # *p* < 0.05 CTRL vs. TNBS.

Figure 9 shows the concentration of colonic Lcn-2. Lcn-2 levels showed a significant increase in the TNBS group compared to the CTRL group (3.12 ± 0.5 vs. 5.63 ± 0.51 ng/mg protein). Nevertheless, while a slight increase was observed with the 5 mg/kg dose of RES, the 10 and 20 mg/kg doses resulted in a marked decrease in the levels of Lcn-2. However, none of these changes reached statistical significance when compared to the TNBS group.

## 3. Materials and Methods

### 3.1. Experimental Animals

Male Wistar–Hannover rats (225–250 g) were obtained (Toxicoop Ltd., Dunakeszi, Hungary) and used in this study. The animals were acclimatized for 7 days prior to the initiation of the experiment. The rats were housed at 20–23 °C with a 12 h light/12 h dark cycle. Animals had ad libitum access to standard laboratory rat chow and water. All procedures were conducted in accordance with the European Community Directive 2010/63/EU on the protection of animals used for scientific purposes and were approved by the Institutional Ethical Committee (I.74-40/2022. MÁB).

### 3.2. Experimental Design

**Figure 10:**
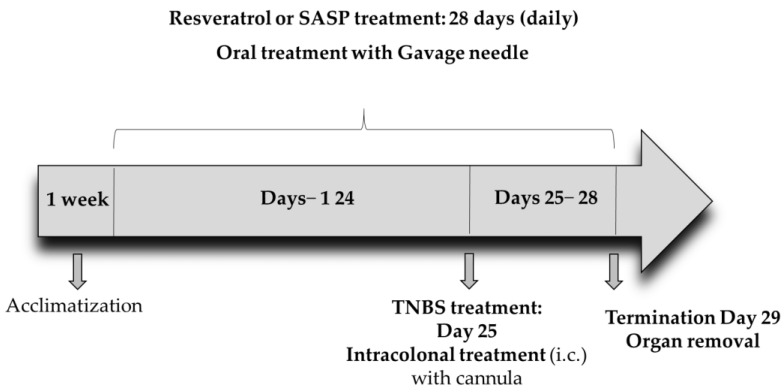
The experimental protocol of the study.

Resveratrol (3,5,4′-trihydroxy-trans-stilbene) was purchased (Sigma-Aldrich, Budapest, Hungary) and was given to the rats 25 days before TNBS injection and then until completion of the experiment for a total of 28 days. Three doses of RES (5, 10, and 20 mg/kg/day) were suspended in tap water [27] and were administered to the animals orally by using an oral gavage needle for 28 consecutive days at the same time every morning at 9 a.m. The final volume of the RES suspension was 1 mL per animal. A total of 65 male rats were included in the experiment. The rats were randomly divided into 7 groups: Group 1: CTRL (no treatment, *n* = 8), Group 2: 50% EtOH (50% EtOH enema, *n* = 6), Group 3: TNBS-induced colitis (TNBS enema, *n* = 12), Group 4: Resveratrol pre-treated group (5 mg/kg) + TNBS-induced colitis rats (*n* = 10), Group 5: Resveratrol pre-treated group (10 mg/kg) +TNBS-induced colitis rats (*n* = 13), Group 6: Resveratrol pre-treated group (20 mg/kg) + TNBS-induced colitis rats (*n* = 10), Group 7: SASP (sulfasalazine) + TNBS-induced colitis rats (*n* = 6) (Table 1). SASP was used as a positive control and was dissolved in physiological saline (0.9%). SASP was administered orally to the rats for 28 days (50 mg/kg/day). On the 25th day of the experiment, colonic inflammation was induced as previously described [28]. Briefly, the rats were fasted for 18 h before intracolonic (i.c.) administration of TNBS in a dose of 10 mg/rat, dissolved in 50% EtOH. TNBS was purchased from Sigma-Aldrich (Budapest, Hungary). The procedure was carried out with an 8 cm-long polyethylene cannula, under thiopental anesthesia (intraperitoneal (i.p.) 40 mg/kg). Then, i.c.-treated animals were kept in a vertical position for one minute to inhibit intracolonic dripping. Finally, the rats were returned to their cages with free access to food and water. The estimated time interval between the oral RES administration and TNBS challenge was approximately 3 h. The rats’ general conditions were assessed daily. The rats were euthanized (thiopental, i.p. 100 mg/kg, Tiobarbital Braun, 0.5 g, B. Braun Medical SA, Barcelona, Spain) 72 h after TNBS administration and the distal 8 cm portion of the colon was dissected, opened in longitudinal direction and washed with ice-cold physiological saline. Photographs of the colonic sections (Panasonic Lumix DMC-TZ6, Panasonic Co., Kadoma, Osaka, Japan) were taken to determine macroscopic inflammation. Colonic tissues were frozen in liquid nitrogen, then pulverized in liquid nitrogen and kept at −80 °C until biochemical measurements. The experimental protocol is presented in Figure 10. All outcome evaluations, including macroscopic, histological, and immunofluorescence analyses, were performed by investigators blinded to the treatment groups. Colonic tissues were obtained and subjected to immunofluorescence staining and histological examination (*n* = 3 animals per group).

### 3.3. Macroscopic Evaluation of the Lesions

The extent of macroscopically apparent inflammation, ulceration, and tissue necrosis were determined in a randomized manner from the color images using proprietary computerized planimetry software (Stat_2_1_1, developed in our laboratory, Szeged, Hungary), based on planimetrics. With this software, the inflamed and the total area of the colon segments were outlined. Then the area of macroscopically visible mucosal damage was calculated and expressed as the percentage (%) of the inflamed area compared to the total 8 cm colonic segment. Through evaluation, we considered the following changes in the colon: redness, hemorrhage, necrosis, and ulcers [29].

### 3.4. Haematoxylin and Eosin Staining

Tissue samples for histology were taken 2–3 cm from the anus and fixed in 4% buffered formaldehyde at 4 °C overnight. Tissues were placed into histology cassettes, dehydrated through ascending grades of ethanol (70–100%) to xylene and embedded in melted paraffin (60–62 °C) in a Leica TP1020 (Leica Biosystems, Wetzlar, Germany) tissue processor. Sections were cut transversely at 5 µm thickness and processed for standard haematoxylin and eosin staining with Leica ST5010 Autostainer (Leica Biosystems, Wetzlar, Germany). Following staining, the tissue was mounted using a permanent mounting medium (CV Ultra, Leica). Sections were observed and photographed with a Zeiss Axio Imager.Z2 light microscope equipped with a Digital Microscopy Camera AxioCam ICc 5 (Zeiss, Oberkochen, Germany) [30,31].

### 3.5. Fluorescent Immunohistochemistry

For immunofluorescence, paraffin sections from different groups were immunostained with TNF-α, TNFR1, or TNFR2. Briefly, after blocking in Tris(hydroxymethyl)aminomethane-buffered saline (TBS) containing 1% bovine serum albumin (BSA) and 10% normal goat serum, the sections were incubated overnight with anti-TNF-α (rabbit polyclonal IgG; ab6671, Abcam, Cambridge, UK, 1:50), anti-TNFR1 (rabbit polyclonal, SAB4502988, Sigma-Aldrich, Budapest, Hungary, 1:100), or anti-TNFR2 (rabbit polyclonal, SAB4502989, Sigma-Aldrich, Budapest, Hungary, 1:200) primary antibodies at 4 °C. After washing in TBS with 0.025% TPBS

riton X-100, sections were incubated with anti-rabbit Alexa Fluor 488 (A11008, Invitrogen, Thermo Fisher Scientific, Waltham, MA, USA, 1:200) secondary antibody for 1 h at room temperature. Negative controls were performed by omitting the primary antibodies when no immunoreactivity was observed. Immunostained sections were mounted in Fluoroshield^TM^ with DAPI Mounting Medium (Sigma-Aldrich, Budapest, Hungary), and observed and photographed with a Zeiss Imager Z.2 fluorescent microscope equipped with an Axiocam 506 mono camera (Zeiss, Jena, Germany) [32].

### 3.6. Determination of Lipocalin-2 and TNF-α Levels in the Colon by ELISA

An equal amount of samples was weighed and homogenized on ice with Handheld homogenizer D1000 (Benchmark Scientific, New Jersey, MA, USA) in pH 7.4 phosphate-buffer saline (PBS). Then, samples were centrifuged at 3000 rpm, 20 min, 4 °C. Parameters were measured according to the instructions and protocols of the manufacturer, and optical densities (OD) were measured at 450 nm (Benchmark Microplate reader, Bio-Rad, Hercules, CA, USA). Concentrations of TNF-α were expressed as pg/mg protein and Lcn-2 as ng/mg protein, respectively.

### 3.7. Protein Determination

Protein concentrations were measured by the BCA Protein Assay kit (Thermo Fisher Scientific, Waltham, MA, USA) according to the instructions (27).

### 3.8. Data Representation and Statistical Analysis

Results are shown as mean ± S.E.M. Statistical analysis was performed using one-way ANOVA followed by Holm–Sidak post-hoc test with SigmaPlot 12.0 software (Systat Software Inc., San Jose, CA, USA). *p* ≤ 0.05 was considered as significant [33].

## 4. Discussion

IBD is a chronic inflammatory syndrome, which affects the gastrointestinal tract with inflammation and ulceration. Several polyphenolic compounds with antioxidant and anti-inflammatory properties, such as RES, are currently under intensive investigation as possible adjunctive or self-contained treatment alternatives in IBD [31]. This study demonstrates that long-term RES treatment can mitigate colonic injury induced by TNBS and reduce the levels of inflammatory parameters.

In this study, TNBS-induced colitis model was used, which is one of the most widely established and reproducible experimental models of IBD, particularly CD. TNBS induces a Th1-mediated immune response, characterized by transmural inflammation and ulceration that closely mimics several pathological features of human IBD. Therefore, findings in this chemically induced model are considered to be of translational relevance to human disease [24,34,35]. The advantages of the TNBS-induced colitis model lie in its well-characterized immunopathological features, including increased cytokine production, immune cell infiltration, and mucosal damage, making it suitable for studying the molecular mechanisms of inflammation and for evaluating potential therapeutic agents, such as RES [24,36,37].

In the present study, we used oral doses of 5, 10, and 20 mg/kg/day of RES for 4 weeks to investigate its effects in TNBS-induced colitis. Based on our results, 10 mg/kg dose of RES appeared to be the most effective with respect to ulcer healing. Our findings show evidence for the anti-inflammatory effect of RES; therefore, this compound may be a promising therapeutic option against IBD.

As a natural polyphenol, RES has several protective effects and a number of studies have reported its molecular mechanisms of action [7,38,39]. RES has a direct radical scavenging and antioxidant effect resulting from its molecular structure [40]. Furthermore, RES participates in various signaling pathways to exert immunomodulatory effects, which reduce the production of pro-inflammatory factors.

Here we demonstrated that 28 days of RES pre-treatment has a protective effect on TNBS colitis in male rats. To determine the protective effect of different doses of RES, we analyzed the degree of inflammation induced by TNBS. Our results clearly showed that the administration of 10 mg/kg RES significantly diminished the extent of ulcers in rats compared to the TNBS-induced colitis group. However, the lowest and highest doses of RES in our current study did not appear to be effective. As a possible explanation, it is conceivable that RES has a hormetic response in different doses [41,42]. Although our findings suggest a possible hormetic response to RES, this interpretation remains speculative, as we did not conduct dedicated experiments to characterize the dose–response relationship. This concept is supported by earlier studies demonstrating biphasic dose effects of polyphenols, including RES, in both in vitro and in vivo models [43,44]. Nevertheless, further studies including a broader range of doses and dose–response analysis are warranted to establish a definitive hormetic effect. Yildiz et al. [26] also showed that i.p. administered RES for 5 days significantly reduced the severity of inflammation in the colonic tissue of TNBS induced colitis rats. Additionally, Martin et al. showed that RES has protective effects in TNBS-induced acute colitis [27]. Furthermore, it was found that RES treatment significantly relieved the symptoms of IBD in a dextran sodium sulfate (DSS)-induced colitis mouse model [45]. Zhou et al. reported that the anti-inflammatory effect of RES is mediated through inhibition of the NF-κB pathway [46]. Based on our findings, long-term RES supplementation appears to be more effective in promoting ulcer healing. The protection of the effective dose of RES was as efficient as a currently used therapeutic agent, SASP. In accordance with our results, Das et al. [47] also verified that the administration of SASP significantly suppressed the severity of inflammation. Our histological results supported the macroscopic evaluation of the lesions. RES at a concentration of 10 mg/kg substantially attenuated the morphological damage in colon tissues caused by TNBS. Yao et al. also found that RES significantly improves histological outcomes and provides therapeutic benefits in a mouse model of colitis [48].

In addition to the antioxidant properties of RES, it should be mentioned that RES has NF-κB transcription factor downregulating effect [49,50,51]. Previous studies reported that RES suppressed the phosphorylation of NF-κB and improved inflammation by regulating the SIRT1/NF-κB pathway [52,53]. NF-κB has a stimulating effect on the transcription of key pro-inflammatory cytokine genes, such as TNF-α. Our findings have underpinned that TNF-α concentration was significantly higher in the TNBS rats compared to the CTRL group. However, 28 days of 10 mg/kg dose of RES treatment significantly decreased the expression of TNF-α which contributed to the attenuation of the colonic damage. Other data showed that 20 mg/kg RES supplementation was able to diminish TNF-α level in chronic DSS-induced colitis [54]. In a mouse model of IBD, RES reduced the expression of pro-inflammatory cytokines and significantly decreased disease activity index [55]. Additionally, a clinical study demonstrated a significant decrease in the level of TNF-α in patients with mild to moderate UC after RES supplementation [56]. Zhang et al. [57] found that RES supplementation for 14 days also reduced the levels of TNF-α in DSS-treated mice.

Our immunohistochemical findings confirm the molecular results representing intensified immunolabeling of TNF-α in the TNBS group, while an attenuation can be observed in the 10 mg/kg dose of RES pre-treated group compared to controls. Increased TNF-α expression can lead to mucosal barrier dysfunction, increase inflammation and worsen the prognosis of IBD [58]. Similar changes were observed in TNFRs immunoreactivity; however, a definite difference was revealed between the immunolabeling of two TNFRs even in control conditions. TNFR2 presence is more pronounced than TNFR1 in controls and especially explicit in the ganglia of the myenteric plexus. Other studies also reported differences in TNF receptor distribution between healthy and type 1 diabetic rats, which were associated with chronic low-grade inflammation [59,60]. Although previous studies have examined the effects of RES on inflammatory signaling pathways and TNF-α in IBD models, our study adds a novel layer by specifically characterizing the expression of TNF receptors (TNFR1 and TNFR2), using immunofluorescence in a TNBS-induced colitis model. This allows for a better understanding of receptor-level modulation by RES treatment in this particular experimental context. To our knowledge, this is the first study to examine TNFR1 and TNFR2 protein expression at the tissue level in a TNBS-induced rat model treated with oral RES over a 4-week period. In the present study, we did not specifically investigate the exact effect of RES on TNFRs; however, based on the current literature, we suppose that the following factors may play a role in the observed phenomenon potentially through pathways involving NF-κB [61], Akt [62], and ERK [63] factors.

Lcn-2 is involved in the pathogenesis of IBD and is considered a biochemical marker of intestinal inflammation. It is shown that the levels of Lcn-2 correlate with the activity of the disease [64]. Our results show that Lcn-2 concentration was significantly higher in TNBS-induced colitis. Similarly, another study showed that Lcn-2 expression is increased in the colonic tissues of patients with active IBD [65]. Moreover, a recent study has demonstrated that Lcn-2 concentration was significantly increased in the colonic tissue of DSS-treated mice [66]. The administration of 10 mg/kg and 20 mg/kg doses of RES markedly reduced the levels of Lcn-2; however, the differences compared to the TNBS group did not show statistical significance.

## 5. Conclusions

In this study, we showed that long-term RES supplementation with RES exerted a beneficial anti-inflammatory effect in the TNBS-induced colitis rat model. RES treatment reduced the expression of TNF-α and modulated the tissue-level immunoreactivity of its receptors, TNFR1 and TNFR2. These findings suggest that RES may influence colonic inflammation through a dual mechanism targeting both cytokines and their receptors. While our results support the potential of RES as an adjunctive strategy in experimental IBD, further studies are warranted to clarify the specific roles of TNFR signaling in this process and to validate these effects in other models.

## 6. Limitations

It is important to note that our data are based on immunofluorescence visualization and provide correlative evidence for TNFR modulation. Further studies using quantitative techniques such as Western blot or qPCR are warranted to elucidate the precise mechanisms involved. Moreover, based on the effects induced by the RES doses applied in the present study, a hormetic dose–response effect can be presumed; however, confirming this assumption requires further investigations.

## Figures and Tables

**Table 1:** The experimental groups of the study.

No.	Group	Treatment
1.	CTRL	no treatment
2.	EtOH	50% EtOH enema
3.	TNBS-induced colitis	TNBS dissolved in 50% ethanol
4.	5 mg/kg resveratrol	RES 5 mg/kg + TNBS dissolved in 50% ethanol
5.	10 mg/kg resveratrol	RES 10 mg/kg + TNBS dissolved in 50% ethanol
6.	20 mg/kg resveratrol	RES 20 mg/kg + TNBS dissolved in 50% ethanol
7.	positive control (SASP)	SASP + TNBS dissolved in 50% ethanol

## Data Availability

The datasets used and/or analyzed during the current study are available from the corresponding author on reasonable request.

## List of Abbreviations

BCA	bicinchoninic acid
BSA	bovine serum albumin
CD	Crohn’s disease
CTRL	control, no treatement
DSS	dextran sodium sulfate
ELISA	enzyme-linked immunosorbent assay
EtOH	ethanol
IBD	inflammatory bowel disease
Lcn-2	lipocalin-2
NF-κB	nuclear factor kappa B
NGAL	neutrophil gelatinase-associated lipocalin
OD	optical density
PBS	phosphate-buffer saline
RES	resveratrol, 3,5,4′-trihydroxy-trans-stilbene
SASP	sulfasalazine
TBS	Tris(hydroxymethyl)aminomethane-buffered saline
TNBS	2,4,6-trinitrobenzene sulfonic acid
TNF-α	tumor necrosis factor-alpha
TNFR	TNF-α receptor
UC	ulcerative colitis
